# Improvement of Post‐Thaw Quality and In Vivo Fertility of Simmental Bull Spermatozoa Using Ferulic Acid

**DOI:** 10.1002/vms3.70064

**Published:** 2024-10-18

**Authors:** Mobin Afsar, Ali Soleimanzadeh, Amir Khaki, Esmail Ayen

**Affiliations:** ^1^ Department of Theriogenology Faculty of Veterinary Medicine Urmia University Urmia Iran; ^2^ Department of Clinical Sciences Faculty of Veterinary Medicine Amol University of Special Modern Technologies Amol Iran

**Keywords:** cryopreservation, ferulic acid, semen quality, Simmental bulls

## Abstract

**Background:**

Artificial insemination and semen cryopreservation have significantly improved the quality and quantity of cattle production. Through cryopreserved semen and artificial insemination, top‐breeding bull sperm can be used to inseminate thousands of cows worldwide.

**Objectives:**

Our study aimed to determine the effect of adding ferulic acid (FA) to a Tris‐based semen extender on frozen and thawed Simmental bull sperm.

**Methods:**

Semen samples were collected from three Simmental bulls. Pooled Simmental semen (*n* = 34 ejaculations) were diluted with a Tris‐base extender containing varying FA concentrations (0.1, 0.15, 0.25, 0.35, and 0.45 mM). After the samples were frozen and thawed, the samples were tested for malondialdehyde (MDA), total antioxidant capacity (TAC), superoxide dismutase (SOD), glutathione peroxidase (GPx), total motility, progressive motility, motility characteristics, and plasma membrane functionality.

**Results:**

The control and the groups with the best FA concentrations, 0.25 and 0.35, were compared for in vivo fertility. Fifty‐one cows were inseminated 24 h after the onset of oestrus. A rectal examination was used to diagnose pregnancies at least 60 days after fertilization. Results showed that adding FA‐0.45, FA‐0.35, FA‐0.25, and FA‐0.15 to the semen of Simmental bulls improved total and progressive motility, motility characteristics, and plasma membrane functionality. It also increased GPx and TAC levels, reducing MDA and DNA damage after freezing. The addition of FA did not affect SOD values. The fertility rate in the FA‐0.25 and FA‐0.35 groups was higher than in the control group, 35.29%, with rates of 76.47% and 70.58%, respectively.

**Conclusions:**

In conclusion, adding FA (0.15, 0.25, 0.35, and 0.45 mM) to Tris‐based semen extenders can improve the quality parameters of cryopreserved Simmental bull semen and increase in vivo fertility using 0.25 and 0.35 concentrations of FA.

## Graphical abstract

Our study revealed that adding ferulic acid to semen extenders improved sperm preservation by reducing DNA damage and lipid peroxidation, while enhancing TAC, GPx, plasma membrane functionality and motility characteristics. Ferulic acid (0.25 and 0.35 mM) can also enhance the fertility of cryopreserved Simmental bull semen after insemination.

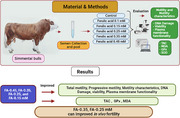

**Abbreviations**
ALH: amplitude of lateral head displacement
AO: acridine orange
BCF: beat‐cross frequency
FA: ferulic acid
GPx: glutathione peroxidase
H&E: haematoxylin and eosin
LIN: linearity
MDA: malondialdehyde
PM: progressive motility
PMF: plasma membrane functionality
ROS: reactive oxygen species
SOD: superoxide dismutase)
STR: straightness
TAC: total antioxidant capacity
TM: total motility
VAP: average path velocity
VCL: curvilinear velocity
VSL: straight‐line velocity

## 1 Introduction

Cryopreservation of semen is an essential method for the long‐term preservation of sperm and is widely used in cattle breeding for genetic advancement through artificial insemination (Ahmed, Andrabi, and Jahan 2016; Andrabi et al. 2008). Sperm that have been frozen and thawed have intact motility apparatus, active mitochondria, integral plasma, and acrosomal membranes (Bag et al. 2004), as well as unbroken DNA (Waterhouse et al. 2010), making them optimal for reproduction. However, increased reactive oxygen and oxidative stress due to cryopreservation can harm spermatozoa, causing them to lose motility, rupture structural membranes and become less fertile, which affects their ability to fertilize (Chatterjee and Gagnon 2001; Najafi et al. 2014).

Lipid peroxidation (LPO) is the result of interactions between reactive oxygen species (ROS) and the sperm plasmalemma (Malekifard, Delirezh, and Soleimanzadeh 2018; Nourian et al. 2017; Soleimanzadeh et al. 2018a; Storey 1997). It increases oxidative stress, damaging sperm structures and functions such as motility, plasmalemma and acrosomal integrity in bull sperm. This oxidative stress is due to an imbalance between ROS production and natural antioxidants' defence mechanisms, impairing mitochondrial function, DNA integrity and sperm metabolism in bulls (Chaveiro et al. 2006). By preserving sperm at lower temperatures, various techniques have been used to extend sperm lifespans by reducing their motility and metabolism (Allai et al. 2018). Several studies have been conducted on the antioxidants used in animal semen (El‐Sheshtawy, El‐Sisy, and El‐Nattat 2008; Izanloo et al. 2021, 2022; Kumar et al. 2015; Parvizi Alan et al. 2024; Sheikholeslami et al. 2020; Soleimanzadeh and Saberivand 2013; Soleimanzadeh, Saberivand, and Ahmadi 2014; Soleimanzadeh et al. 2018c). Studies have shown that incorporating various enzymatic and non‐enzymatic antioxidants into semen extenders can have different results (Varghese et al. 2015). Antioxidants are critical for sperm motility and integrity, as well as for sperm metabolism and function (Soleimanzadeh, Malekifard, and Kabirian 2017; Soleimanzadeh et al. 2020a; Soleimanzadeh et al. 2018a; Soleimanzadeh, Mohammadnejad, and Ahmadi 2018b). They also shield cells from oxidative damage, thereby enhancing the semen's ability to fertilize and preserve it (El‐Sheshtawy, El‐Sisy, and El‐Nattat 2008; Ezz et al. 2017; Kadirvel, Kumar, and Kumaresan 2009).

Ferulic acid (FA) is a naturally occurring material in plant cell walls, mainly found in fruits, vegetables and seeds (Itagaki et al. 2009). It plays a biological role in neutralizing ROS and free radicals, which is mainly related to its antioxidant activity (Koh 2012). Due to its low toxicity, as demonstrated in animal models, FA is considered a safe food additive in many European countries. It inhibits the activity of oxidases, increases the activity of antioxidant enzymes and shows high scavenging activity against hydrogen peroxide, superoxide, hydroxyl radicals and nitrogen dioxide‐free radicals (Ou and Kwok 2004). Studies have shown that FA has the highest antioxidant activity compared to other phenolic acids with comparable molecular structures (Kanski et al. 2002). Furthermore, FA protects neurons from oxidative stress‐induced apoptosis after cerebral ischaemia and has been shown to improve rat sperm parameters in vivo (Zheng and Zhang 1997) and human sperm motility in vitro (Roy et al. 2014). Technological advances have made it possible to preserve semen for artificial insemination in various species, especially bulls. However, despite these advances, there are still gaps in our knowledge and technology in this area. Semen survival rates after thawing remain low and vary widely among breeding bulls. A potential solution to this problem is the use of natural antioxidants, such as FA, to improve the freezing survival of Simmental sperm. Currently, there are no reports on the effects of FA on Simmental bull sperm during cryopreservation. This study aims to determine the optimal dose of FA and its effects on the in vivo fertility and quality of Simmental bull sperm during cryopreservation to improve their cryo survival after freezing and thawing.

## 2 Materials and Methods

### 2.1 Chemicals

All required chemicals were provided by Merck (Darmstadt, Germany) and Sigma (St. Louis, Missouri, USA).

### 2.2 Semen Collection and Processing

**FIGURE 1:**
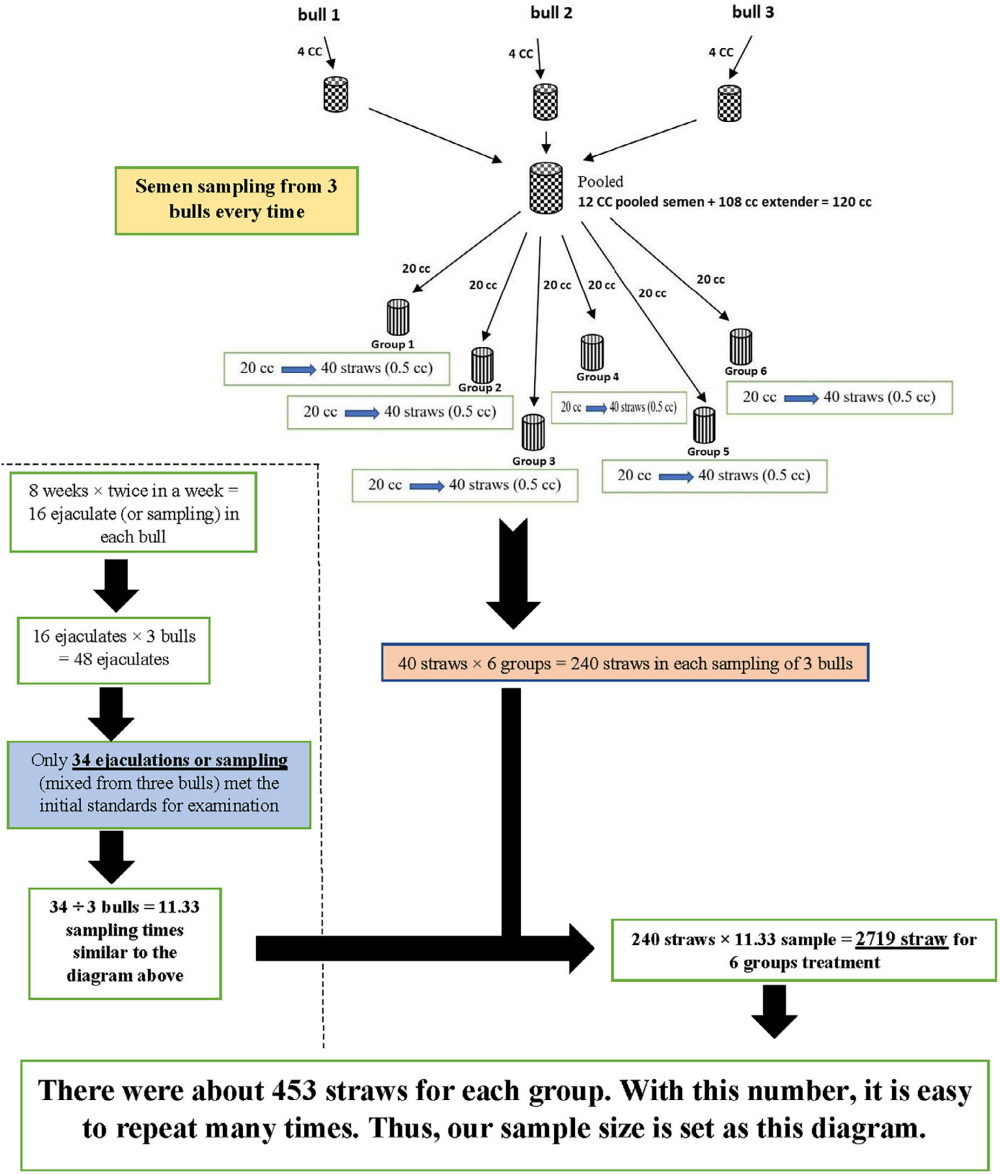
Sampling of Simmental bulls.

All animals received identical nutrition and management throughout the study, with semen collected twice a week using an artificial vagina (AV). Within 8 weeks, 3 Simmental bulls with known fertility produced 34 ejaculations (Figure 1). The samples were subjected to a quality assessment. They met specific criteria, including a volume between 2 and 6 mL, a concentration of more than 1 × 10^9^ cells/mL, a total motility of more than 50% and an abnormal morphology of no more than 20% per ejaculation.

The Tris‐based extender (Tris: 2.66 g per 100 mL, citric acid: 1.47 g per 100 mL, glucose: 0.63 g per 100 mL, egg yolk: 20% (v/v), glycerin: 7% v/v, penicillin: 1 mg per 100 mL, streptomycin: 1 mg per 100 mL, pH: 6.8) (Ramazani et al. 2023a) was used for semen dilution, and the final concentration was 15 × 10^6^ spermatozoa/mL achieved using a single extender from the list below. Group control and five additional groups were formed by adding different amounts of antioxidants to the diluent. FA was at concentrations of 0.1, 0.15, 0.25, 0.35, and 0.45 mM (FA‐0.1, FA‐0.15, FA‐0.25, FA‐0.35, and FA‐0.45) (Pei et al. 2018) added to the Tris base extender. After 1 week, the frozen samples were thawed and thawed in a water bath at 38°C to be ready for analysis (Ramazani et al. 2023a).

### 2.3 Semen Analysis

#### 2.3.1 Assessment of Total and Progressive Motility and Motility Characteristics

The CASA system (Test Sperm 3.2; Videotest, Moscow, St. Petersburg) was used to measure the parameters of sperm motility. Ten microlitres of thawed sperm were used for the analysis, and at least 500 sperm were analysed in 5 microscopic fields. The system evaluated various parameters, including average path velocity (VAP), curvilinear velocity (VCL), straight‐line velocity (VSL), linearity (LIN), the amplitude of lateral head displacement (ALH), straightness (STR) and beat cross frequency (BCF) (Table 1; Ramazani et al. 2023b).

**TABLE 1:** Parameter settings for the CASA.

Parameter	Setting
Frame rate	60 Hz
Duration of capture	1 s
Stage temperature rate	37°C
Minimum cell size	5 pixels
Cell size	5 pixels
Minimum contrast	80
Cell intensity	70 pixels
Chamber type	Slide‐coverslip (22 × 22 mm^2^)
Volume per slide	7 µL
Chamber depth	≈20 µm
Minimum number of field analysis	500 cells
Sample dilution	20 × 10^6^
Image type	Phased contrast

#### 2.3.2 Assessment of Viability

The eosin–nigrosin staining technique was utilized to precisely and quickly assess sperm viability (Organisation 1999). Two volumes of 1% eosin and one volume of sperm were used, and the mixture was examined with a light microscope (Olympus Optics Co., model CHT) at 400× magnification. Viable sperm remained colourless, whereas non‐viable sperm turned red (Ramazani et al. 2023b).

#### 2.3.3 Sperm Plasma Membrane Functionally (PMF)

Hypoosmotic Swelling Test (HOST) was used to determine whether sperm had intact membranes. The test involved diluting 10 µL of sperm in 100 µL of a hypoosmotic solution containing 1.35 g fructose and 0.73 g sodium citrate. The sperm were then incubated at 37°C and examined for PMF using a contrast phase microscope (Olympus, BX41, Tokyo, Japan) at 400× magnification. Two hundred straight or curved sperm were counted to determine the percentage of sperm with PMF (Khan and Ijaz 2007; Ramazani et al. 2023a).

#### 2.3.4 Assessment of DNA Damage

The acridine orange (AO) staining technique was used to assess DNA damage and identify segments of denatured double‐stranded DNA. Carnoy's fixative, which consists of methanol and acetic acid in a 1:3 ratio, was applied to a thick smear and left for 2 h. This was followed by air drying for 5 min at room temperature. The swab was then immersed in a stock solution containing 1 mg AO and 1000 mL of distilled water and stored in the dark at 4°C for 5 min. Sperm showing red or yellow fluorescence were deemed damaged and abnormal, whereas those exhibiting green fluorescence were classified as having normal DNA (Soleimanzadeh et al. 2020b).

### 2.4 Evaluation of the Antioxidant Activity of Enzymes

Biochemical analysis of the semen involved the use of 15 straws (one for each extender). The sperm was thawed and centrifuged at 25°C and 1600 × *g* for 5 min to extract its enzymes. The combination was then centrifuged at 25°C and 4000 × *g* for 30 min (Ramazani et al. 2023a).

#### 2.4.1 Total Antioxidant Capacity (TAC), Glutathione Peroxidase (GPx) and Superoxide Dismutase (SOD) Evaluation

Seven straws were used to assess oxidative enzymes in each experimental group. After thawing, 120 µL of sperm was centrifuged at 25°C and 1600 × *g* for 5 min (Ramazani et al. 2023a).

The concentrations of TAC, GPx, and SOD activities in semen samples were analysed using kits (Naxifer, Nagpix and Nasdox, respectively) from Navand Salamat Company in Urmia, Iran. TAC value was reported in mmol/L, whereas the GPx value was reported in mU/mL. On the other hand, SOD activity in seminal plasma was expressed in U/mL (Ramazani et al. 2023a).

#### 2.4.2 Amounts of Malondialdehyde (MDA)

The MDA test kit (Nalondi Lipid Peroxidation Assay Kit; Navand Salamat Company, Urmia, Iran) was used to measure oxidative stress. The MDA concentration was determined at a wavelength of 523 nm using a spectrophotometer (Thermo Fisher Scientific; Waltham, MA, USA) (Ramazani et al. 2023b) and a standard curve, which is expressed as nmol/mg protein.

### 2.5 Fertility Rate After Artificial Insemination

Before use for in vivo fertility insemination, the quality of thawed sperm was assessed after 24 h of storage in LN_2_. For the in vivo fertility tests, adult wheeled cattle in their second or third lactation were utilized. The reproductive systems of the 51 adult wheeled cattle (aged 4–8 years) were clinically normal and displayed genuine oestrus symptoms (without synchronization). The mucus discharge and the decrease in milk production were indicators of oestrus. After approximately 24 h following the onset of oestrus, all experimental inseminations were finished. On the basis of their improved results after thawing, the control and supplement groups (FA‐0.25 and FA‐0.35; best groups) were selected, and fertility levels were compared. A rectal pregnancy diagnosis was made at least 60 days after the insemination of each cow by the inseminator (Ramazani et al. 2023b).

### 2.6 Statistical Analysis

Statistical analysis was performed using the Statistical Package for the Social Sciences software, version 26 (SPSS Inc., Chicago, IL, USA). Before comparison, the Kolmogorov–Smirnov test was first performed for normality. Then, the Kruskal–Walli's test was used, and the multiple tests were corrected by the Bonferroni method for the non‐normal distribution of the parameters. One‐way ANOVA was used to determine the significance of the differences between the comparison groups. Tukey's post hoc analysis was used to identify significant differences between individual groups. The in vivo fertility rate was analysed using the Chi‐square test. A statistically significant *p* value was defined as ≤0.05.

## 3 Results

### 3.1 Sperm Total and Progressive Motility and Motility Characteristics

As shown in Table 2, total motility and progressive motility of sperm are improved by FA‐0.45, FA‐0.35, FA‐0.25, and FA‐0.15 (*p* ≤ 0.05). However, there is no significant difference between the control group and FA‐0.1‐treated samples (*p* > 0.05). VAP, VCL, VSL, and LIN values are also significantly improved in the FA‐0.45, FA‐0.35, FA‐0.25, and FA‐0.15 groups compared to the control group (*p* ≤ 0.05; Table 2). However, there is no significant difference in ALH, STR, and BCF values during storage (*p* > 0.05).

**TABLE 2:** Sperm total and progressive motility and motility characteristics of frozen‐thawed Simmental semen after adding different ferulic acid (FA) concentrations in semen extender (mean ± SEM).

Analysis	Control	FA‐0.1	FA‐0.15	FA‐0.25	FA‐0.35	FA‐0.45
Total motility (%)	59.87 ± 1.23^c^	58.73 ± 1.18^c^	67.18 ± 1.34^b^	73.41 ± 1.43^a^	73.22 ± 1.68^a^	68.46 ± 1.72^b^
Progressive motility (%)	17.51 ± 1.92^c^	18.56 ± 1.73^c^	27.45 ± 1.69^b^	36.81 ± 1.48^a^	37.35 ± 1.14^a^	25.02 ± 1.80^b^
VAP (µm/s)	30.43 ± 1.79^c^	30.25 ± 1.54^c^	36.41 ± 1.73^b^	41.87 ± 1.25^a^	41.54 ± 1.17^a^	35.17 ± 1.62^b^
VCL (µm/s)	35.23 ± 1.62^c^	35.51 ± 1.32^c^	40.71 ± 1.32^b^	45.67 ± 1.41^a^	44.19 ± 1.26^a^	41.32 ± 1.83^b^
VSL (µm/s)	19.59 ± 1.16^c^	21.65 ± 1.43^c^	25.11 ± 1.36^b^	31.75 ± 1.69^a^	29.32 ± 1.53^a^	25.64 ± 1.25^b^
LIN (%)	33.59 ± 1.57^c^	35.27 ± 1.83^c^	40.03 ± 1.79^b^	46.96 ± 1.67^a^	45.68 ± 1.42^a^	39.59 ± 1.53^b^
ALH (µm/s)	2.41 ± 0.19^a^	2.65 ± 0.14^a^	2.39 ± 0.17^a^	2.58 ± 0.24^a^	2.46 ± 0.13^a^	2.53 ± 0.10^a^
STR (%)	66.51 ± 1.34^a^	65.78 ± 1.51^a^	66.73 ± 1.09^a^	66.45 ± 1.84^a^	66.32 ± 1.25^a^	65.84 ± 1.69^a^
BCF (Hz)	2.49 ± 0.19^a^	2.43 ± 0.20^a^	2.66 ± 0.14^a^	2.32 ± 0.15^a^	2.51 ± 0.17^a^	2.34 ± 0.12^a^

*Note*: FA‐0.1, Tris‐based extender + ferulic acid (0.1 mM); FA‐0.15, Tris‐based extender + ferulic acid (0.15 mM); FA‐0.25, Tris‐based extender + ferulic acid (0.25 mM); FA‐0.35, Tris‐based extender + ferulic acid (0.35 mM); FA‐0.45, Tris‐based extender + ferulic acid (0.45 mM). Different superscripts within the row demonstrate significant differences (*p *≤ 0.05).

Abbreviations: ALH, amplitude of lateral head displacement; BCF, beat‐cross frequency; LIN, linearity; STR, straightness; VAP, average path velocity; VCL, curvilinear velocity; VSL, straight‐line velocity.

### 3.2 Sperm Viability, Plasma Membrane Functionally, and DNA Damage

The viability and HOST analyses in Table 3 show that the FA‐0.45, FA‐0.35, FA‐0.25, and FA‐0.15 groups have higher values than the control group (*p *≤ 0.05). However, the FA‐0.1 group showed no significant difference (*p* > 0.05). DNA damage analysis using AO staining indicates that the FA‐0.45, FA‐0.35, FA‐0.25, and FA‐0.15 groups have less DNA damage than the control group (*p* ≤ 0.05; Table 3). On the other hand, the FA‐0.1 and control groups have more DNA damage.

**TABLE 3:** Effect of different concentrations of ferulic acid (FA) supplementation on DNA damage, viability and plasma membrane functionality (PMF) in frozen‐thawed Simmental sperm (mean ± SEM).

Analysis	Control	FA‐0.1	FA‐0.15	FA‐0.25	FA‐0.35	FA‐0.45
Viability (%)	58.26 ± 1.27^c^	60.47 ± 1.22^c^	66.17 ± 1.81^b^	71.69 ± 1.34^a^	72.10 ± 1.39^a^	65.39 ± 1.02^b^
Sperm plasma membrane functionality (%)	52.84 ± 1.75^c^	52.19 ± 1.95^c^	57.32 ± 1.46^b^	63.51 ± 1.69^a^	61.47 ± 1.25^a^	58.22 ± 1.53^b^
DNA damage (%)	10.37 ± 0.26^c^	9.25 ± 0.19^c^	8.61 ± 0.33^b^	4.73 ± 0.26^a^	4.85 ± 0.13^a^	7.15 ± 0.45^b^

*Note*: FA‐0.1, Tris‐based extender + ferulic acid (0.1 mM); FA‐0.15, Tris‐based extender + ferulic acid (0.15 mM); FA‐0.25, Tris‐based extender + ferulic acid (0.25 mM); FA‐0.35, Tris‐based extender + ferulic acid (0.35 mM); FA‐0.45, Tris‐based extender + ferulic acid (0.45 mM). Different superscripts within the row demonstrate significant differences (*p *≤ 0.05).

### 3.3 Evaluation of Antioxidant Activities

**FIGURE 2:**
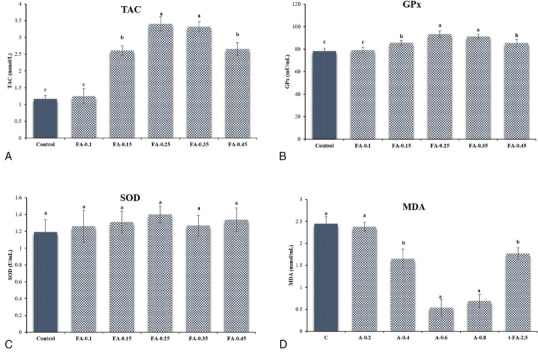
(A) Total antioxidant capacity (TAC) of frozen‐thawed Simmental bull semen after supplementing different concentrations of ferulic acid to the semen extender. FA‐0.1: Tris‐based extender + ferulic acid (0.1 mM); FA‐0.15: Tris‐based extender + ferulic acid (0.15 mM); FA‐0.25: Tris‐based extender + ferulic acid (0.25 mM); FA‐0.35: Tris‐based extender + ferulic acid (0.35 mM); FA‐0.45: Tris‐based extender + ferulic acid (0.45 mM). Superscripts demonstrate significant differences (*p* ≤ 0.05). (B) glutathione peroxidase (GPx) of frozen‐thawed Simmental bull semen after supplementing different concentrations of ferulic acid to the semen extender. FA‐0.1: Tris‐based extender + ferulic acid (0.1 mM); FA‐0.15: Tris‐based extender + ferulic acid (0.15 mM); FA‐0.25: Tris‐based extender + ferulic acid (0.25 mM); FA‐0.35: Tris‐based extender + ferulic acid (0.35 mM); FA‐0.45: Tris‐based extender + ferulic acid (0.45 mM). Superscripts demonstrate significant differences (*p* ≤ 0.05). (C) Superoxide dismutase (SOD) of frozen‐thawed Simmental bull semen after supplementing different concentrations of ferulic acid to the semen extender. FA‐0.1: Tris‐based extender + ferulic acid (0.1 mM); FA‐0.15: Tris‐based extender + ferulic acid (0.15 mM); FA‐0.25: Tris‐based extender + ferulic acid (0.25 mM); FA‐0.35: Tris‐based extender + ferulic acid (0.35 mM); FA‐0.45: Tris‐based extender + ferulic acid (0.45 mM). Superscripts demonstrate significant differences (*p* ≤ 0.05). (D) Lipid peroxidation (MDA) of frozen‐thawed Simmental bull semen after supplementing different concentrations of ferulic acid to the semen extender. FA‐0.1: Tris‐based extender + ferulic acid (0.1 mM); FA‐0.15: Tris‐based extender + ferulic acid (0.15 mM); FA‐0.25: Tris‐based extender + ferulic acid (0.25 mM); FA‐0.35: Tris‐based extender + ferulic acid (0.35 mM); FA‐0.45: Tris‐based extender + ferulic acid (0.45 mM). Superscripts demonstrate significant differences (*p* ≤ 0.05).

Figure 2 shows that the addition of FA significantly increased the TAC (Figure 2A) and GPx (Figure 2B) activity in the FA‐0.45, FA‐0.35, FA‐0.25, and FA‐0.15 groups compared to the control group (*p* ≤ 0.05). However, there is no significant change in TAC and GPx values in FA‐0.1 and control groups (*p* > 0.05). Notably, the SOD (Figure 2C) values show no effect of FA addition (*p* > 0.05). The addition of FA‐0.45, FA‐0.35, FA‐0.25, and FA‐0.15 also decreased MDA (Figure 2D) values compared to the other groups (*p* ≤ 0.05).

### 3.4 Assessment of In Vivo Fertility Rate

Table 5 presents the findings of fertility tests conducted in vivo, which reveal that the pregnancy rate is higher in the FA‐0.25 and FA‐0.35 groups when compared to the control group (*X* ^2^ (2, *N* = 51) = 7.07, *p* = 0.02). However, there is no difference in fertility between the FA‐0.25 and FA‐0.35 groups (Table 4).

**TABLE 4:** Comparison of fertility rate of Simmental semen cryopreserved.

Extender	Number of conceptions	Pregnancy rate (%)	*p* value	Chi‐square statistic
Control (no:17)	6	35.29^b^	0.02	7.07
FA‐0.25 (no:17)	13	76.47^a^		
FA‐0.35 (no:17)	12	70.58^a^		

*Note*: FA‐0.25, Tris‐based extender + ferulic acid (0.25 mM); FA‐0.35, Tris‐based extender + ferulic acid (0.35 mM). Different superscripts within the column demonstrate significant differences (*p *≤ 0.05).

Abbreviation: FA, ferulic acid.

## 4 Discussion

The study shows that adding FA to the extender can improve the semen of thawed Simmental bulls in many ways. Two previous studies have also demonstrated that adding antioxidants to the extender can increase the freezing ability of Simmental bull semen (Parvizi Alan et al. 2024; Tahmasbian, Ayen, and Khaki 2022). Bull semen does not have enough antioxidants to protect it from oxidative damage caused by freezing. Therefore, to improve sperm viability after freezing, exogenous antioxidants must be added. Previous researches have shown that supplementing sperm expanders with various antioxidants can improve these parameters (Ejaz et al. 2014; Ramazani et al. 2023a; Ramazani et al. 2023b; Saeed et al. 2016; Soleimanzadeh et al. 2020a; Turaja et al. 2019)

Sperm motility is essential, as shown by Kasai et al. (2002) and Soleimanzadeh et al. (2020a). It represents the energy state of spermatozoa (Quintero‐Moreno, Rigau, and Rodrıguez‐Gil 2004). Adding antioxidants to semen extenders can improve sperm vitality by reducing ROS levels (Sharafi, Zhandi, and Akbari Sharif 2015). Sperm motility is a crucial measure of sperm quality during fluid preservation, which ROS can negatively affect (Cerolini et al. 2000). Previous studies have demonstrated that supplementing the extender of Simmental bull with antioxidants 0.4 and 0.6 mM rutin (Farjami, Soleimanzadeh, and Ayen 2024) and 20.00 µmol epigallocatechin‐3‐gallate (Parvizi Alan et al. 2024) could enhance total and progressive motility during cryopreservation. According to Zheng and Zhang (1997), FA can improve human sperm motility in both fertile and infertile individuals. Their study found that taking FA supplements can boost parameters such as total and progressive motility; however, it does not affect ALH, BCF, and STR. Similarly, Pei et al. (2018) showed how FA can improve boar sperm motility after cryopreservation. Recent studies also suggest that adding FA to the semen extender for goat bucks can enhance semen motility when stored in the refrigerator (Zhang et al. 2023).

Research has shown that acute necrotic changes can occur in sperm due to osmotic imbalances during cryopreservation. After thawing, a significant proportion of remaining sperm may suffer sublethal damage that could lead to apoptosis‐like cell death and shorten their lifespan (Said, Gaglani, and Agarwal 2010). These results highlight the need for additional research to develop more effective strategies to maintain sperm quality and improve cryopreservation procedures for semen storage (Ortega‐Ferrusola et al. 2009). Using the correct extender and maintaining the storage temperature at the proper level is essential to improving sperm viability and maintaining plasma membrane functions. Ansari et al. (2017) reported that there is a possibility of permanent functional impairment of sperm viability due to cryopreservation, partly due to a decrease in the proportion of intact acrosomes and total acrosin activity. Sperm exposed to low temperatures are susceptible to cold shock damage (Akhtar et al. 2022; Dziekońska et al. 2022). Extender solutions often contain antioxidants and protective chemicals to counteract the negative effects of storage on semen quality, such as reduced viability and functionality of the plasma membrane (Izanloo et al. 2021, 2022). Mohammadi, Hosseinchi Gharehaghaj, and Novin (2024) showed that adding 10 mM *trans*‐FA to a buffalo bull semen extender could improve sperm viability, PMF and DNA damage during cryopreservation. Pei et al. (2018) showed that FA can improve motility, PMF, mitochondrial activity and acrosomal integrity of frozen and thawed boar semen. Furthermore, FA has been shown to enhance sperm viability and kinematics in diabetic rats (Roy et al. 2014). The present study's results show that FA succeeded in increasing the percentage of viable cells and maintaining PMF in cryopreserved Simmental bull semen.

Male infertility is associated with numerous factors, including genetic abnormalities, hormonal imbalances, problems with spermatogenesis, poor sperm quality and sperm DNA fragmentation (Esteves et al. 2012). Kumaresan et al. (2017) found a significant association between dairy bull fertility and DNA damage. Comparable reports on the importance of sperm DNA integrity for fertility and/or semen quality exist for other livestock, such as stallions, boars and rams (Neuhauser et al. 2019; Peña et al. 2019; Peris‐Frau et al. 2019). Bulls with lower reproductive potential exhibit various DNA integrity parameters in their ejaculates (Bochenek, Smorąg, and Pilch 2001). In one study, Farjami, Soleimanzadeh, and Ayen (2024) showed that adding 0.4 and 0.6 mM rutin to the extender could improve sperm DNA damage during Simmental bull semen preservation. In this study, comparing the FA‐supplemented extender group with the control group showed a significant improvement in sperm DNA integrity. Similarly, research has demonstrated that epigallocatechin‐3‐gallate can reduce DNA damage in Simmental bull semen during cryopreservation (Parvizi Alan et al. 2024). Furthermore, Tahmasbian, Ayen, and Khaki (2022) found that hesperidin can improve sperm DNA integrity after cryopreservation.

Studies by Partyka, Łukaszewicz, and Niżański (2012) and Kasimanickam et al. (2006) have shown that the activity of antioxidant enzymes in sperm is directly related to the quality and quantity of sperm. The freeze‐thaw process of cryopreservation can lead to the production of ROS, which can damage plasma membranes and reduce the activity of antioxidant enzymes (Alvarez and Storey 1995). Osmotic stress during cryopreservation may also contribute to oxidative damage. The excess of ROS caused by stress can damage the mitochondrial membrane and affect sperm quality (Ball 2008). FA has been found to stimulate the activity of antioxidant enzymes and improve sperm quality (Ou and Kwok 2004). Combining FA with apigenin has been shown to improve boar semen quality (Pei et al. 2018). Previous research has shown that the antioxidant status during cryopreservation can be improved by adding 10 mM *trans*‐FA to the extender of buffalo bull semen (Mohammadi, Hosseinchi Gharehaghaj, and Novin 2024). Different concentrations of FA were found to increase the antioxidant enzymes TAC and GPx and decrease MDA levels. However, our study did not find a significant improvement in SOD values when comparing the FA extender with the control group.

The preservation process can cause physical and chemical stress to sperm, including the formation of intracellular ice crystals, osmotic changes and oxidative stress (Khan et al. 2021). To maintain optimal reproductive health, stress during preservation must be reduced (Agarwal et al. 2022). Our study showed that adding 0.25‐ and 0.35‐mM FA could increase the reproductive rate. The high integrity of the plasma membrane has been found to impact sperm viability significantly and results in improved conception rates. Studies by Tariq et al. (2020) and Khalid, El‐Gohary, and Ahmed (2020) have also suggested that adding carboxylated poly‐l‐lysine and cysteine to sperm cryopreservation can improve in vivo fertility, activate the antioxidant system and improve sperm motility. The addition of quercetin to bull semen was found to improve progressive motility, membrane integrity, acrosomes and DNA after thawing (Ahmed et al. 2019). Moreover, using antioxidants during cryopreservation has been shown to increase the reproductive rate of buffalo sperm (Ramazani et al. 2023b).

## 5 Conclusion

Our study found that adding 0.15, 0.25, 0.35 and 0.45 mM of FA to semen extenders can significantly improve several semen quality parameters. Specifically, FA enhanced total and progressive motility, motility characteristics, viability, plasma membrane functionality and reduced DNA damage. Additionally, it increased TAC and GPx levels, which are crucial for maintaining sperm integrity during cryopreservation. Notably, adding FA at concentrations of 0.25 and 0.35 mM to Tris‐based semen extenders showed the most promise, as it significantly improved the in vivo fertility of cryopreserved Simmental bull semen post‐insemination. This suggests that FA at these concentrations not only preserves semen quality during storage but also enhances the likelihood of successful fertilization after thawing and insemination. These findings highlight the potential of FA as a valuable supplement in semen extenders for improving cryopreservation outcomes and artificial insemination in cow reproduction programmes.

## Author Contributions

Ali Soleimanzadeh and Amir Khaki were involved in the idea, design, data collecting, statistical analysis and paper preparation. Mobin Afsar, Ali Soleimanzadeh, Amir Khaki and Esmail Ayen all contributed to the study's supervision, as well as the manuscript's drafting. The final version was accepted for submission by all writers.

## Ethics Statement

The Animal Ethics Committee of Urmia University, located in Urmia, Iran, approved the protocols for the care and management of animals in accordance with its guidelines. The study was conducted under approval number IR‐UU‐AEC‐3/58.

## Conflicts of Interest

The authors declare no conflicts of interest.

## Data Availability

The data supporting this study's findings are available on request from the corresponding author. However, due to privacy or ethical restrictions, the data are not publicly available.

## References

[vms370064-bib-0001] Agarwal, A. , I. Maldonado Rosas , C. Anagnostopoulou , et al. 2022. “Oxidative Stress and Assisted Reproduction: A Comprehensive Review of Its Pathophysiological Role and Strategies for Optimizing Embryo Culture Environment.” Antioxidants 11, no. 3: 477. 10.3390/antiox11030477. 35326126 PMC8944628

[vms370064-bib-0002] Ahmed, H. , S. M. H. Andrabi , and S. Jahan . 2016. “Semen Quality Parameters as Fertility Predictors of Water Buffalo Bull Spermatozoa During Low‐Breeding Season.” Theriogenology 86, no. 6: 1516–1522. 10.1016/j.theriogenology.2016.05.010. 27321805

[vms370064-bib-0003] Ahmed, H. , S. Jahan , M. M. Salman , and F. Ullah . 2019. “Stimulating Effects of Quercetin (QUE) in Tris Citric Acid Extender on Post Thaw Quality and In Vivo Fertility of Buffalo (*Bubalus bubalis*) Bull Spermatozoa.” Theriogenology 134: 18–23. 10.1016/j.theriogenology.2019.05.012. 31112913

[vms370064-bib-0004] Akhtar, M. F. , Q. Ma , Y. Li , et al. 2022. “Effect of Sperm Cryopreservation in Farm Animals Using Nanotechnology.” Animals 12, no. 17: 2277. 10.3390/ani12172277. 36077996 PMC9454492

[vms370064-bib-0007] Allai, L. , A. Benmoula , M. da Silva Maia , B. Nasser , and B. El Amiri . 2018. “Supplementation of Ram Semen Extender to Improve Seminal Quality and Fertility Rate.” Animal Reproduction Science 192: 6–17. 10.1016/j.anireprosci.2018.03.019. 29615291

[vms370064-bib-0008] Alvarez, J. G. , and B. T. Storey . 1995. “Differential Incorporation of Fatty Acids Into and Peroxidative Loss of Fatty Acids From Phospholipids of Human Spermatozoa.” Molecular Reproduction and Development 42, no. 3: 334–346. 10.1002/mrd.1080420311. 8579848

[vms370064-bib-0009] Andrabi, S. M. H. , M. S. Ansari , N. Ullah , and M. Afzal . 2008. “Effect of Non‐Enzymatic Antioxidants in Extender on Post‐Thaw Quality of Buffalo (*Bubalus bubalis*) Bull Spermatozoa.” Pakistan Veterinary Journal 28, no. 4: 159–162.

[vms370064-bib-0010] Ansari, M. S. , B. A. Rakha , R. Ejaz , N. Ullah , and S. Akhter . 2017. “Melatonin Supplementation in Extender Enhances the Post Thaw Quality of Buffalo Bull Spermatozoa.” Pakistan Journal of Zoology 49, no. 1: 163–167.

[vms370064-bib-0011] Bag, S. , A. Joshi , S. M. K. Naqvi , and J. P. Mittal . 2004. “Effect of Post‐Thaw Incubation on Sperm Kinematics and Acrosomal Integrity of Ram Spermatozoa Cryopreserved in Medium‐Sized French Straws.” Theriogenology 62, no. 3–4: 415–424. 10.1016/j.theriogenology.2003.10.018. 15225998

[vms370064-bib-0012] Ball, B. A. 2008. “Oxidative Stress, Osmotic Stress and Apoptosis: Impacts on Sperm Function and Preservation in the Horse.” Animal Reproduction Science 107, no. 3–4: 257–267. 10.1016/j.anireprosci.2008.04.014. 18524506

[vms370064-bib-0013] Bochenek, M. , Z. Smorąg , and J. Pilch . 2001. “Sperm Chromatin Structure Assay of Bulls Qualified for Artificial Insemination.” Theriogenology 56, no. 4: 557–567. 10.1016/S0093-691X(01)00588-X. 11572437

[vms370064-bib-0014] Cerolini, S. , A. Maldjian , P. Surai , and R. Noble . 2000. “Viability, Susceptibility to Peroxidation and Fatty Acid Composition of Boar Semen During Liquid Storage.” Animal Reproduction Science 58, no. 1–2: 99–111. 10.1016/S0378-4320(99)00035-4. 10700648

[vms370064-bib-0015] Chatterjee, S. , and C. Gagnon . 2001. “Production of Reactive Oxygen Species by Spermatozoa Undergoing Cooling, Freezing, and Thawing.” Molecular Reproduction and Development: Incorporating Gamete Research 59, no. 4: 451–458. 10.1002/mrd.1052. 11468782

[vms370064-bib-0016] Chaveiro, A. , L. Machado , A. Frijters , B. Engel , and H. Woelders . 2006. “Improvement of Parameters of Freezing Medium and Freezing Protocol for Bull Sperm Using Two Osmotic Supports.” Theriogenology 65, no. 9: 1875–1890. 10.1016/j.theriogenology.2005.10.017. 16310842

[vms370064-bib-0017] Dziekońska, A. , N. M. Neuman , K. K. Burdal , A. Wiszniewska‐Łaszczych , and M. Bogdaszewski . 2022. “The Effect of Different Extenders on the Quality Characteristics of European Red Deer Epididymal Sperm Stored at 5°C.” Animals 12, no. 19: 2669. 10.3390/ani12192669. 36230410 PMC9559589

[vms370064-bib-0018] Ejaz, R. , M. S. Ansari , B. A. Rakha , et al. 2014. “Arachidic Acid in Extender Improves Post‐Thaw Parameters of Cryopreserved Nili‐Ravi Buffalo Bull Semen.” Reproduction in Domestic Animals 49, no. 1: 122–125. 10.1111/rda.12239. 24112366

[vms370064-bib-0019] El‐Sheshtawy, R. I. , G. A. El‐Sisy , and W. S. El‐Nattat . 2008. “Use of Selected Amino Acids to Improve Buffalo Bull Semen Cryopreservation.” Global Veterinaria 2, no. 4: 146–150.

[vms370064-bib-0020] Esteves, S. C. , A. Zini , N. Aziz , J. G. Alvarez , E. S. Sabanegh Jr. , and A. Agarwal . 2012. “Critical Appraisal of World Health Organization's New Reference Values for Human Semen Characteristics and Effect on Diagnosis and Treatment of Subfertile Men.” Urology 79, no. 1: 16–22. 10.1016/j.urology.2011.08.003. 22070891

[vms370064-bib-0021] Ezz, M. A. , A. E. Montasser , M. Hussein , et al. 2017. “The Effect of Cholesterol Loaded Cyclodextrins on Post‐Thawing Quality of Buffalo Semen in Relation to Sperm DNA Damage and Ultrastructure.” Reproductive Biology 17, no. 1: 42–50. 10.1016/j.repbio.2016.12.001. 28041717

[vms370064-bib-0022] Farjami, A. , A. Soleimanzadeh , and E. Ayen . 2024. “Evaluation of Co‐Supplementation of Rutin in Simmental Bull Semen Extender: Evaluation of Kinetic Parameters, Sperm Quality.” Veterinary Research & Biological Products 37, no. 1: 79–88.

[vms370064-bib-0023] Itagaki, S. , T. Kurokawa , C. Nakata , et al. 2009. “In Vitro and In Vivo Antioxidant Properties of Ferulic Acid: A Comparative Study With Other Natural Oxidation Inhibitors.” Food Chemistry 114, no. 2: 466–471. 10.1016/j.foodchem.2008.09.073.

[vms370064-bib-0024] Izanloo, H. , A. Soleimanzadeh , M. N. Bucak , M. Imani , and M. Zhandi . 2021. “The Effects of Varying Concentrations of Glutathione and Trehalose in Improving Microscopic and Oxidative Stress Parameters in Turkey Semen During Liquid Storage at 5°C.” Cryobiology 101: 12–19. 10.1016/j.cryobiol.2021.07.002. 34245722

[vms370064-bib-0025] Izanloo, H. , A. Soleimanzadeh , M. N. Bucak , M. Imani , and M. Zhandi . 2022. “The Effects of Glutathione Supplementation on Post‐Thawed Turkey Semen Quality and Oxidative Stress Parameters and Fertilization, and Hatching Potential.” Theriogenology 179: 32–38. 10.1016/j.theriogenology.2021.11.010. 34823059

[vms370064-bib-0026] Kadirvel, G. , S. Kumar , and A. Kumaresan . 2009. “Lipid Peroxidation, Mitochondrial Membrane Potential and DNA Integrity of Spermatozoa in Relation to Intracellular Reactive Oxygen Species in Liquid and Frozen‐Thawed Buffalo Semen.” Animal Reproduction Science 114, no. 1–3: 125–134. 10.1016/j.anireprosci.2008.10.002. 19010614

[vms370064-bib-0027] Kanski, J. , M. Aksenova , A. Stoyanova , and D. A. Butterfield . 2002. “Ferulic Acid Antioxidant Protection Against Hydroxyl and Peroxyl Radical Oxidation in Synaptosomal and Neuronal Cell Culture Systems In Vitro: Structure‐Activity Studies.” Journal of Nutritional Biochemistry 13, no. 5: 273–281. 10.1016/S0955-2863(01)00215-7. 12015157

[vms370064-bib-0028] Kasai, T. , K. Ogawa , K. Mizuno , et al. 2002. “Relationship Between Sperm Mitochondrial Membrane Potential, Sperm Motility, and Fertility Potential.” Asian Journal of Andrology 4, no. 2: 97–104. 12085099

[vms370064-bib-0029] Kasimanickam, R. , K. D. Pelzer , V. Kasimanickam , W. S. Swecker , and C. D. Thatcher . 2006. “Association of Classical Semen Parameters, Sperm DNA Fragmentation Index, Lipid Peroxidation and Antioxidant Enzymatic Activity of Semen in Ram‐Lambs.” Theriogenology 65, no. 7: 1407–1421. 10.1016/j.theriogenology.2005.05.056. 16188307

[vms370064-bib-0030] Khalid, K. A. , A. E. El‐Gohary , and A. M. A. Ahmed . 2020. “Raising the Efficiency of Lemon Trees to Produce Essential Oil by Exogenous Cysteine Under Various Soil Structures.” Journal of Essential Oil Bearing Plants 23, no. 1: 194–203. 10.1080/0972060X.2020.1736646.

[vms370064-bib-0031] Khan, I. M. , Z. Cao , H. Liu , et al. 2021. “Impact of Cryopreservation on Spermatozoa Freeze‐Thawed Traits and Relevance Omics to Assess Sperm Cryo‐Tolerance in Farm Animals.” Frontiers in Veterinary Science 8: 609180. 10.3389/fvets.2021.609180. 33718466 PMC7947673

[vms370064-bib-0032] Khan, M. , and A. Ijaz . 2007. “Assessing Undiluted, Diluted and Frozen‐Thawed Nili‐Ravi Buffalo Bull Sperm by Using Standard Semen Assays.” Italian Journal of Animal Science 6: 784–787. 10.4081/ijas.2007.s2.784.

[vms370064-bib-0033] Koh, P.‐O. 2012. “Ferulic Acid Prevents the Cerebral Ischemic Injury‐Induced Decrease of Akt and Bad Phosphorylation.” Neuroscience Letters 507, no. 2: 156–160. 10.1016/j.neulet.2011.12.012. 22200499

[vms370064-bib-0034] Kumar, P. , D. Kumar , P. Sikka , and P. Singh . 2015. “Sericin Supplementation Improves Semen Freezability of Buffalo Bulls by Minimizing Oxidative Stress During Cryopreservation.” Animal Reproduction Science 152: 26–31. 10.1016/j.anireprosci.2014.11.015. 25497424

[vms370064-bib-0035] Kumaresan, A. , A. Johannisson , E. M. Al‐Essawe , and J. M. Morrell . 2017. “Sperm Viability, Reactive Oxygen Species, and DNA Fragmentation Index Combined Can Discriminate Between Above‐and Below‐Average Fertility Bulls.” Journal of Dairy Science 100, no. 7: 5824–5836. 10.3168/jds.2016-12484. 28478003

[vms370064-bib-0036] Malekifard, F. , N. Delirezh , and A. Soleimanzadeh . 2018. “Modulatory Effect of Pioglitazone on Sperm Parameters and Oxidative Stress, Apoptotic and Inflammatory Biomarkers in Testes of Streptozotocin‐Induced Diabetic Rats.” International Journal of Medical Laboratory 5, no. 1: 19–34. http://ijml.ssu.ac.ir/article‐1‐172‐en.html.

[vms370064-bib-0037] Mohammadi, T. , M. Hosseinchi Gharehaghaj , and A. A. Novin . 2024. “Effects of Apigenin and Trans‐Ferulic Acid on Microscopic and Oxidative Stress Parameters in the Semen of Water Buffalo Bulls During Cryopreservation.” Cryobiology 115: 104868. 10.1016/j.cryobiol.2024.104868. 38423495

[vms370064-bib-0038] Najafi, A. , H. D. Kia , H. Mohammadi , et al. 2014. “Different Concentrations of Cysteamine and Ergothioneine Improve Microscopic and Oxidative Parameters in Ram Semen Frozen With a Soybean Lecithin Extender.” Cryobiology 69, no. 1: 68–73. 10.1016/j.cryobiol.2014.05.004. 24854868

[vms370064-bib-0039] Neuhauser, S. , H. Bollwein , M. Siuda , and J. Handler . 2019. “Effects of Different Freezing Protocols on Motility, Viability, Mitochondrial Membrane Potential, Intracellular Calcium Level, and DNA Integrity of Cryopreserved Equine Epididymal Sperm.” Journal of Equine Veterinary Science 82: 102801. 10.1016/j.jevs.2019.102801. 31732114

[vms370064-bib-0006] Nourian, A. , A. Soleimanzadeh , A. S. Jalali , and G. Najafi . 2017. “Effects of Bisphenol‐S Low Concentrations on Oxidative Stress Status and In Vitro Fertilization Potential in Mature Female Mice.” Veterinary Research Forum 8, no. 4: 341–345. http://www.ncbi.nlm.nih.gov/pubmed/29326794%0Ahttp://www.pubmedcentral.nih.gov/articlerender.fcgi?artid=PMC5756255. 29326794 PMC5756255

[vms370064-bib-0041] Ortega‐Ferrusola, C. , B. M. García , J. M. Gallardo‐Bolanos , et al. 2009. “Apoptotic Markers Can be Used to Forecast the Freezeability of Stallion Spermatozoa.” Animal Reproduction Science 114, no. 4: 393–403. 10.1016/j.anireprosci.2008.10.005. 19019584

[vms370064-bib-0042] Ou, S. , and K. Kwok . 2004. “Ferulic Acid: Pharmaceutical Functions, Preparation and Applications in Foods.” Journal of the Science of Food and Agriculture 84, no. 11: 1261–1269. 10.1002/jsfa.1873.

[vms370064-bib-0043] Partyka, A. , E. Łukaszewicz , and W. Niżański . 2012. “Effect of Cryopreservation on Sperm Parameters, Lipid Peroxidation and Antioxidant Enzymes Activity in Fowl Semen.” Theriogenology 77, no. 8: 1497–1504. 10.1016/j.theriogenology.2011.11.006. 22225691

[vms370064-bib-0005] Parvizi Alan, A. , E. Ayen , A. Khaki , and A. Soleimanzadeh . 2024. “Epigallocatechin‐3‐Gallate Affects the Quality of Fresh and Frozen‐Thawed Semen of Simmental Bull by Two Different Cryopreservation Methods.” Veterinary Research Forum 15, no. 7: 369–377. 39257464 10.30466/vrf.2024.2000236.3852 PMC11383200

[vms370064-bib-0045] Pei, Y. , L. Yang , L. Wu , et al. 2018. “Combined Effect of Apigenin and Ferulic Acid on Frozen‐Thawed Boar Sperm Quality.” Animal Science Journal 89, no. 7: 956–965. 10.1111/asj.13009. 29708294

[vms370064-bib-0046] Peña, S. T. Jr. , B. Gummow , A. J. Parker , and D. B. B. P. Paris . 2019. “Antioxidant Supplementation Mitigates DNA Damage in Boar (*Sus scrofa domesticus*) Spermatozoa Induced by Tropical Summer.” PLoS ONE 14, no. 4: e0216143. 10.1371/journal.pone.0216143. 31039205 PMC6490925

[vms370064-bib-0047] Peris‐Frau, P. , M. Álvarez‐Rodríguez , A. Martín‐Maestro , et al. 2019. “Comparative Evaluation of DNA Integrity Using Sperm Chromatin Structure Assay and Sperm‐Ovis‐Halomax During In Vitro Capacitation of Cryopreserved Ram Spermatozoa.” Reproduction in Domestic Animals 54: 46–49. 10.1111/rda.13519. 31625230

[vms370064-bib-0048] Quintero‐Moreno, A. , T. Rigau , and J. E. Rodrıguez‐Gil . 2004. “Regression Analyses and Motile Sperm Subpopulation Structure Study as Improving Tools in Boar Semen Quality Analysis.” Theriogenology 61, no. 4: 673–690. 10.1016/S0093-691X(03)00248-6. 14698057

[vms370064-bib-0049] Ramazani, N. , F. M. Gharebagh , A. Soleimanzadeh , et al. 2023b. “Reducing Oxidative Stress by κ‐Carrageenan and C60HyFn: The Post‐Thaw Quality and Antioxidant Status of Azari Water Buffalo Bull Semen.” Cryobiology 111: 104–112. 10.1016/j.cryobiol.2023.04.003. 37142111

[vms370064-bib-0050] Ramazani, N. , F. Mahd Gharebagh , A. Soleimanzadeh , et al. 2023b. “The Influence of L‐Proline and Fulvic Acid on Oxidative Stress and Semen Quality of Buffalo Bull Semen Following Cryopreservation.” Veterinary Medicine and Science 9, no. 4: 1791–1802. 10.1002/vms3.1158. 37197763 PMC10357270

[vms370064-bib-0051] Roy, S. , S. K. Metya , N. Rahaman , S. Sannigrahi , and F. Ahmed . 2014. “Ferulic Acid in the Treatment of Post‐Diabetes Testicular Damage: Relevance to the Down Regulation of Apoptosis Correlates With Antioxidant Status Via Modulation of TGF‐β1, IL‐1β and Akt Signalling.” Cell Biochemistry and Function 32, no. 1: 115–124. 10.1002/cbf.2983. 23661600

[vms370064-bib-0052] Saeed, A. M. , H. A. El‐Nagar , W. M. Wafa , and Y. S. Hussein . 2016. “Effect of Coenzyme Q10 as an Antioxidant Added to Semen Extender During Cryopreservation of Buffalo and Cattle Semen.” Journal of Animal and Poultry Production 7, no. 11: 403–408. 10.21608/jappmu.2016.48748.

[vms370064-bib-0053] Said, T. M. , A. Gaglani , and A. Agarwal . 2010. “Implication of Apoptosis in Sperm Cryoinjury.” Reproductive Biomedicine Online 21, no. 4: 456–462. 10.1016/j.rbmo.2010.05.011. 20800544

[vms370064-bib-0054] Sharafi, M. , M. Zhandi , and A. Akbari Sharif . 2015. “Supplementation of Soybean Lecithin‐Based Semen Extender by Antioxidants: Complementary Flowcytometric Study on Post‐Thawed Ram Spermatozoa.” Cell and Tissue Banking 16: 261–269. 10.1007/s10561-014-9458-5. 24907919

[vms370064-bib-0055] Sheikholeslami, S. A. , A. Soleimanzadeh , A. Rakhshanpour , and D. Shirani . 2020. “The Evaluation of Lycopene and Cysteamine Supplementation Effects on Sperm and Oxidative Stress Parameters During Chilled Storage of Canine Semen.” Reproduction in Domestic Animals 55, no. 9: 1229–1239. 10.1111/rda.13770. 32644236

[vms370064-bib-0056] Soleimanzadeh, A. , and A. Saberivand . 2013. “Effect of Curcumin on Rat Sperm Morphology After the Freeze‐Thawing Process.” Veterinary Research Forum 4, no. 3: 185–189. 25653795 PMC4312379

[vms370064-bib-0057] Soleimanzadeh, A. , A. Saberivand , and A. Ahmadi . 2014. “Effect of α‐Tocopherol on Spermatozoa of Rat Semen After the Freeze‐Thawing Process.” Urmia Medical Journal 25, no. 9: 826–834.

[vms370064-bib-0058] Soleimanzadeh, A. , F. Malekifard , and A. R. Kabirian . 2017. “Protective Effects of Hydro‐Alcoholic Garlic Extract on Spermatogenic Disorders in Streptozotocin‐Induced Diabetic C57BL/6 Mice.” Scientific Journal of Kurdistan University of Medical Sciences 22, no. 4: 8–17.

[vms370064-bib-0059] Soleimanzadeh, A. , M. Kian , S. Moradi , and F. Malekifard . 2018a. “Protective Effects of Hydro‐Alcoholic Extract of *Quercus brantii* Against Lead‐Induced Oxidative Stress in the Reproductive System of Male Mice.” Avicenna Journal of Phytomedicine 8, no. 5: 448–456. 30345232 PMC6190249

[vms370064-bib-0060] Soleimanzadeh, A. , L. Mohammadnejad , and A. Ahmadi . 2018b. “Ameliorative Effect of Allium Sativum Extract on Busulfan‐Induced Oxidative Stress in Mice Sperm.” Veterinary Research Forum 9, no. 3: 265–271. 10.30466/vrf.2018.32079. 30357081 PMC6198159

[vms370064-bib-0061] Soleimanzadeh, A. , M. Pourebrahim , N. Delirezh , and M. Kian . 2018c. “Ginger Ameliorates Reproductive Toxicity of Formaldehyde in Male Mice: Evidences for Bcl‐2 and Bax.” Journal of Herbmed Pharmacology 7, no. 4: 259–266. 10.15171/jhp.2018.39.

[vms370064-bib-0062] Soleimanzadeh, A. , M. Kian , S. Moradi , and S. Mahmoudi . 2020a. “Carob (*Ceratonia siliqua* L.) Fruit Hydro‐Alcoholic Extract Alleviates Reproductive Toxicity of Lead in Male Mice: Evidence on Sperm Parameters, Sex Hormones, Oxidative Stress Biomarkers and Expression of Nrf2 and iNOS.” Avicenna Journal of Phytomedicine 10, no. 1: 35–49. 31921606 PMC6941692

[vms370064-bib-0063] Soleimanzadeh, A. , N. Talavi , V. S. Yourdshahi , and M. N. Bucak . 2020b. “Caffeic Acid Improves Microscopic Sperm Parameters and Antioxidant Status of Buffalo (*Bubalus bubalis*) Bull Semen Following Freeze‐Thawing Process.” Cryobiology 95: 29–35. 10.1016/j.cryobiol.2020.06.010. 32590017

[vms370064-bib-0064] Storey, B. T. 1997. “Biochemistry of the Induction and Prevention of Lipoperoxidative Damage in Human Spermatozoa.” Molecular Human Reproduction 3, no. 3: 203–213. 10.1093/molehr/3.3.203. 9237246

[vms370064-bib-0065] Tahmasbian, H. , E. Ayen , and A. Khaki . 2022. “Evaluation of the Effects of Hesperidin on Fresh and Frozen‐Thawed Semen Quality Using Two Different Cryopreservation Methods in Simmental Bull.” Animal Reproduction 19, no. 3: e20220042. 10.1590/1984-3143-ar2022-0042. 36313596 PMC9613352

[vms370064-bib-0066] Tariq, A. , M. Ahmad , S. Iqbal , et al. 2020. “Effect of Carboxylated Poly l‐Lysine as a Cryoprotectant on Post‐Thaw Quality and In Vivo Fertility of Nili Ravi Buffalo (*Bubalus bubalis*) Bull Semen.” Theriogenology 144: 8–15. 10.1016/j.theriogenology.2019.12.012. 31884337

[vms370064-bib-0067] Turaja, K. I. B. , R. S. A. Vega , T. A. Saludes , et al. 2019. “Influence and Total Antioxidant Capacity of Non‐Enzymatic Antioxidants on the Quality and Integrity of Extended and Cryopreserved Semen of Murrah Buffalo (*Bubalus bubalis*).” Philippine Journal of Science 148, no. 4: 619–626.

[vms370064-bib-0068] Varghese, O. , A. J. Dhami , K. K. Hadiya , J. A. Patel , and S. C. Parmar . 2015. “Role of Antioxidants Cysteine and Taurine in Tris Egg Yolk Based Extender for Cryopreservation of Surti Buffalo Semen.” Indian Journal of Animal Reproduction 36, no. 2: 39–45.

[vms370064-bib-0069] Waterhouse, K. E. , A. Gjeldnes , A. Tverdal , et al. 2010. “Alterations of Sperm DNA Integrity During Cryopreservation Procedure and In Vitro Incubation of Bull Semen.” Animal Reproduction Science 117, no. 1–2: 34–42. 10.1016/j.anireprosci.2009.04.011. 19481887

[vms370064-bib-0040] World Health Organization . 1999. WHO Laboratory Manual for the Examination of Human Semen and Sperm‐Cervical Mucus Interaction. Cambridge: Cambridge University Press.

[vms370064-bib-0070] Zhang, F. , S. Han , N. Zhang , J. Chai , and Q. Xiong . 2023. “Effect of Ferulic Acid on Semen Quality of Goat Bucks During Liquid Storage at 17°C.” Animals 13, no. 15: 2469. 10.3390/ani13152469. 37570278 PMC10417205

[vms370064-bib-0071] Zheng, R.‐L. , and H. Zhang . 1997. “Effects of Ferulic Acid on Fertile and Asthenozoospermic Infertile Human Sperm Motility, Viability, Lipid Peroxidation, and Cyclic Nucleotides.” Free Radical Biology and Medicine 22, no. 4: 581–586. 10.1016/S0891-5849(96)00272-9. 9013120

